# Signaling Modulations of miR-206-3p in Tooth Morphogenesis

**DOI:** 10.3390/ijms21155251

**Published:** 2020-07-24

**Authors:** Sanjiv Neupane, Yam Prasad Aryal, Tae-Young Kim, Chang-Yeol Yeon, Chang-Hyeon An, Ji-Youn Kim, Hitoshi Yamamoto, Youngkyun Lee, Wern-Joo Sohn, Jae-Young Kim

**Affiliations:** 1 Department of Biochemistry, School of Dentistry, Kyungpook National University, Daegu 41940, Korea; yamaryal@yahoo.com (Y.P.A.); tae09290@daum.net (T.-Y.K.); yhs2669@naver.com (C.-Y.Y.); ylee@knu.ac.kr (Y.L.); 2 Department of Biochemistry and Cell Biology, Stony Brook University, Stony Brook, NY 11794-5215, USA; 3 Department of Oral and Maxillofacial Radiology, School of Dentistry, Kyungpook National University, Daegu 41940, Korea; chan@knu.ac.kr; 4 Department of Dental Hygiene, College of Health Science, Gachon University, Incheon 21936, Korea; hoho6434@gachon.ac.kr; 5 Department of Histology and Developmental Biology, Tokyo Dental College, Tokyo 101-0061, Japan; hyamamoto@tdc.ac.jp; 6 Pre-Major of Cosmetics and Pharmaceutics, Daegu Haany University, Gyeongsan 38610, Korea; undine75@gmail.com

**Keywords:** epigenetic regulation, tooth crown formation, epithelial-mesenchymal interactions, signaling regulations

## Abstract

MicroRNAs (miRNAs) are a class of naturally occurring small non-coding RNAs that post-transcriptionally regulate gene expression in organisms. Most mammalian miRNAs influence biological processes, including developmental changes, tissue morphogenesis and the maintenance of tissue identity, cell growth, differentiation, apoptosis, and metabolism. The *miR-206-3p* has been correlated with cancer; however, developmental roles of this miRNA are unclear. In this study, we examined the expression pattern and evaluated the developmental regulation of *miR-206-3p* during tooth morphogenesis using ex-vivo culture method. The expression pattern of *miR-206-3p* was examined in the epithelium and mesenchyme of developing tooth germ with stage-specific manners. Perturbation of the expression of *miR-206-3p* clearly altered expression patterns of dental-development–related signaling molecules, including *Axin2, Bmp2, Fgf4, Lef1* and *Shh*. The gene expression complemented with change in cellular events including, apoptosis and proliferation which caused altered crown and pulp morphogenesis in renal-capsule–calcified teeth. Especially, mislocalization of β-Catenin and SMAD1/5/8 were observed alongside dramatic alterations in the expression patterns of *Fgf4* and *Shh*. Overall, our data suggest that the *miR-206-3p* regulate the cellular physiology during tooth morphogenesis through modulation of the Wnt, Bmp, Fgf, and Shh signaling pathways to form proper tooth pulp and crown.

## 1. Introduction

Tooth development is a molecularly and morphologically well-defined process. For decades, gene expression patterns and functions of a range of signaling factors in tooth development have been examined using cells, tissues, and genetically manipulated animals [1]. However, we still do not understand the details of the modulations of the signaling pathways that underlie tooth morphogenesis. The missing link manages the cross-talk among the signaling pathways by modulating the levels of their dental-development–related components, and is known as the key factors for tooth development. It includes paracrine and transcription factors, and needs to be elucidated to understand the detailed molecular mechanisms underlying tooth development and regeneration.

Tooth development progresses through the interactions of dental epithelium and neural-crest–derived mesenchyme, and is mediated by multiple members of the Bmp, Fgf, Shh, and Wnt signaling pathways [1]. These pathways are known to be regulated by activators, inhibitors and mediators for proper tooth morphogenesis [1,2]. Particularly, Wnt signaling components, including Wnt ligands, receptors, transducers, transcription factors, and antagonists are expressed in the dental epithelium and mesenchyme during tooth development in humans and mice [3,4]. In dental epithelium, *Wnt3, Wnt4, Wnt6, Wnt7b, and Wnt10b* are expressed, whereas *Wnt5a* is expressed in the mesenchyme [4] in both mice and humans [3]. During tooth development, the canonical Wnt signaling pathway is activated at multiple stages of tooth morphogenesis, [5]. Modulation of the Wnt signaling results in various tooth phenotypes including, multiple tooth formation, shape changes, developmental arrest, and thin incisors [5]. The activation of the Wnt signaling component β-catenin in the epithelium and mesenchyme results in continuous tooth generation [5], and inhibits the formation of posterior molars [6], respectively. Similarly, other paracrine and transcription factors, including FGF4, LEF1, and BMP4 are involved in the critical stages of tooth development, and perturbation of these factors results in altered proliferation, apoptosis, differentiation or migration of cells, leading to developmental defects, such as tooth agenesis, macrodontia, microdontia, oligodontia, and enamel and dentine defects [7]. However, tooth development has not been sufficiently described and understood only with these signaling molecules, including paracrine and transcription factors, to understand the detailed molecular mechanisms underlying tooth morphogenesis. It is necessary to reveal the fine-tuned signaling regulations with the specific modulators, which would mediate the proper tooth morphogenesis for understanding the precise mechanisms of tooth morphogenesis, especially crown morphogenesis.

MicroRNAs (miRNAs) are 19–25-nt non-coding single-stranded RNAs that modulate gene expression at the post-transcriptional level, and this regulatory mechanism is believed to be important for fine-tuning the cross talk among signaling pathways during development. The developmental roles of miRNA has been demonstrated in various ectodermal organs like skin, hair and teeth [8,9,10]. Deletion of Dicer1 in the mesenchyme results in enamel-free incisors and cuspless molars [11,12,13]. Similarly, *miRNA 135a, miR-224, miR-145* and *miR-143* have been reported to regulate the Bmp signaling [14], enamel mineralization [13,15], and odontoblast differentiation [16], respectively. In swine, *ssc-mir-133b* regulates apoptosis [17] during tooth development. These previous observations suggest that miRNAs would be fine modulators for playing important roles in signaling regulations during tooth morphogenesis in mammals. 

In this study, we selected one of miRNAs, *miR-206-3p*, which shows specific expression pattern in developing tooth germ and skin, and also have role to inhibit adipogenesis [18], but not yet evaluated its developmental function in tooth morphogenesis. In in vitro cell cultivation, miR-206-3p inhibits adipogenesis of adipocyte through silencing c-Met and inactivating the PI3K/Akt signaling pathway [18]. Moreover, PI3K/Akt signaling pathway converge with GSK3β and β-catenin of Wnt signaling [19] suggesting that *miR-206-3p* can modulate Wnt signaling in other model systems including, tooth development. Here, we investigated the precise developmental roles of *miR-206-3p* in modulation of the Wnt signaling, which alters the cross-talks among other signaling pathways during tooth crown and pulp morphogenesis. Therefore, this study aimed to illuminate the modulating roles of *miR-206-3p* in tooth development and broaden our understanding of how miRNAs coordinate developmental signaling pathways during organogenesis.

## 2. Results

### 2.1. miR-206-3p Is Moderately Expressed in the Developing Molar

**Figure 1:**
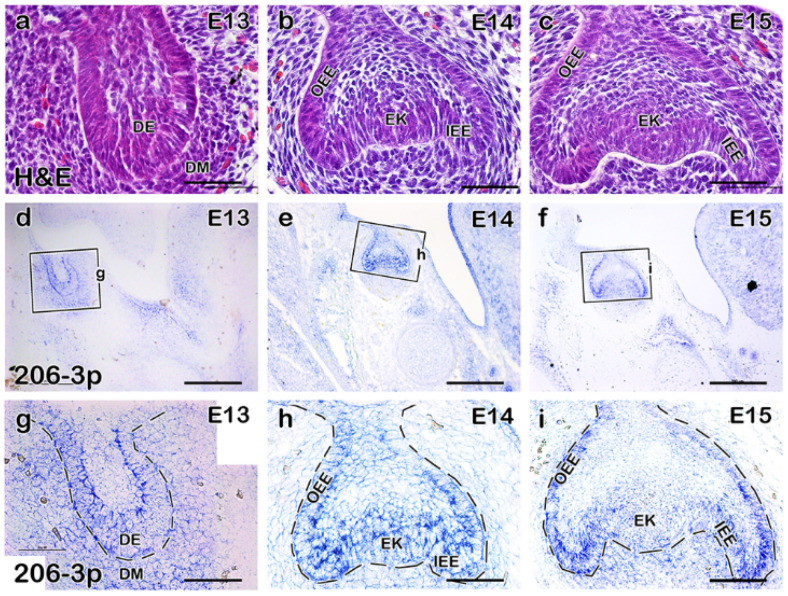
Expression of *miR-206-3p* in the developing tooth. Expression of *miR-206-3p* in the developing tooth. Developing teeth are at the bud and cap stages at E13-E15 (**a**–**c**). Section in situ hybridization using miRCURY LNA^TM^ miRNA Custom Detection Probe for *miR-206-3p* was performed to detect the expression of *miR-206-3p* in the developing tooth at the bud and cap stages (**d**–**i**). Specific expression of *miR-206-3p* was detected in the dental epithelium and condensed mesenchyme at E13 (**d**,**g**). The expression was more restricted to the epithelium, including IEE, OEE, and EK at E14 (**e**,**h**). At E15, the expression was detected in IEE and OEE (**f**,**i**). Schematic diagram showing the expression of *miR-206-3p* (**g**–**i**). DE; dental epithelium, DM; dental mesenchyme, EK; enamel knot, IEE; inner enamel epithelium, OEE; outer enamel epithelium. Rectangular boxes indicate the magnified regions of the developing tooth (**d**–**f**), and the dotted lines indicate the boundary of the epithelium (**g**–**i**). Scale bars 200 µm (**d**–**f**), 50 µm (**a**–**c**,**g**–**i**).

Although specific molecular signaling pathways for tooth development have been described, role of miRNA in tooth development is limited. Therefore, we aimed to identify and determine the developmental roles of miRNA in developing mouse molar. Our pilot screening for miRNA from developing mouse molar at E14 suggested that *miR-206-3p* is highly expressed in developing tooth which led us to select the *miR-206-3p* for further evaluation. The bud-stage (Figure 1a) and cap-stage (Figure 1b,c) developing teeth of E13, and E14/E15 mice, respectively, were used to understand the detailed expression of *miR-206-3p*. Section in situ hybridization using miRCURY LNA^TM^ miRNA Custom Detection Probe for *miR-206-3p* was used to detect the expression of *miR-206-3p* in the developing tooth at the bud and cap stages (Figure 1d–i). 

The expression of *miR-206-3p* was specifically detected in the dental epithelium and condensed mesenchyme at E13 (Figure 1d,g). The expression was more restricted and punctate in the epithelium, including IEE (inner enamel epithelium), OEE (outer enamel epithelium), and EK (enamel knot) at E14 (Figure 1e,h). At the late cap stage (E15), the expression was observed in the IEE and OEE (Figure 1f–i). MicroRNA *miR-206-3p* was also broadly expressed in the dental mesenchyme at E14 and E15 (Figure 1e,f,i,j). IEE and dental mesenchyme are important for hard tissue morphogenesis, including, enamel, and dentine, respectively, as well as dental pulp morphogenesis [20], suggesting that the expression patterns of *miR-206-3p* in developing tooth may provide insight into crown and pulp morphogenesis. 

### 2.2. miR-206-3p Regulates Cell Dynamics Ex Vivo

**Figure 2:**
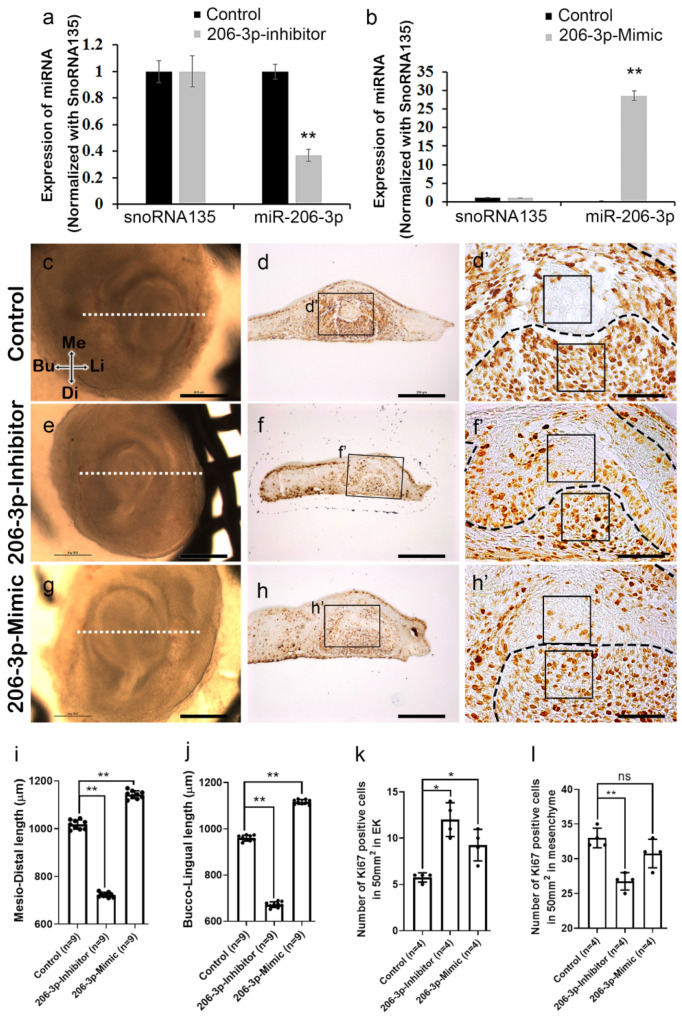
Loss- or gain-of-function of *miR-206-3p* using organ culture. Inhibitor and mimic significantly altered the expression of *miR-206-3p* at E13+36 h cultivated teeth (**a**,**b**). Pre–cap-stage developing teeth at E13 were cultivated for 2 days (**c**,**e**,**g**). The control specimen showed a proper tooth size and structure after 2 days of cultivation (**c**,**i**,**j**). The specimen treated with the inhibitor showed decreased tooth size both bucco-lingually and mesio-distally (**e**,**i**,**j**). The mimic-treated specimen showed a larger tooth structure (**g**,**i**,**j**) after 2 days of cultivation. In comparison with the control (**d**–**d’**,**k**), cellular proliferation increased in both the inhibitor and mimic treated specimen in EK (50 µm^2^) (**f**–**f’**,**h**–**h’**,**k**) and decreased in the mesenchyme (**l**). Bu; buccal, Li; lingual, Me; Mesial, Di; distal, EK; enamel knot. Dotted lines indicate the section levels (**c**,**e**,**g**) and epithelial boundary (**d’**,**f’**,**h’**), and boxes denote the magnified regions (**d**,**f**,**h**) and 50 µm^2^ regions in EK and mesenchyme (d’,f’,h’). ns, * and ** indicate not signigicant, *p* < 0.05 and *p* < 0.01, respectively. *Scale bars* 500 µm (**c**,**e**,**g**), 200 µm (**d**,**f**,**h**), 50 µm (**d’**,**f’**,**h’**).

The inhibitor against *miR-206-3p,* and mimic for *miR-206-3p* were used, respectively to knockdown and increase the function of *miR-206-3p* during in vitro organ cultivation prior to cap stage morphogenesis at E13 (Figure 2a,b). A 36 h cultivation at E13 with the *miR-206-3p* inhibitor and mimic, the expression level of the *miR-206-3p* was evaluated using qPCR (Figure 2a,b), which showed significant decrease, and increase of *miR-206-3p* expression, respectively. When developing teeth at E13 were cultivated for 2 days, the control specimen (*n* = 9) showed proper tooth size and structure after the cultivation (Figure 2c). The inhibitor-and mimic treated specimen (*n* = 9) showed decreased and increased tooth size respectively in both mesio-distally and bucco-lingually (Figure 2e,g,i–j), suggesting that there is alteration in, either cell proliferation or apoptosis. The inhibitor and mimic were fluorescently labelled to monitor the transfections (Figure S1). Epithelial morphogenesis in mouse molar relies on asymmetrical growth, differential cell proliferation, and dynamic cell rearrangement [21,22,23,24] that ensure proper crown and pulp development [25]. In control (*n* = 4), cellular proliferation was observed mostly in the epithelium and mesenchyme, except the EK (Figure 2d–d’,k–l). In contrast, the inhibitor and mimic treated specimen (*n* = 4) showed increased cell proliferation in the EK (Figure 2f–f’,h–h’, k). In addition, inhibitor treated specimen (*n* = 4) showed reduced cell proliferation in the mesenchyme (Figure 2f–f’,l). On the other hand, mimic treated specimen showed no significant proliferation in the mesenchyme (Figure 2h–h’,l). 

These observations of cell proliferation suggested that decrease in tooth germ size after treatment with inhibitor is directly related to the decreased mesenchymal cell proliferation. The increased cell proliferation in EK of both the inhibitor and mimic treated specimen suggest that EK morphogenesis is altered which affects the EK signaling, ultimately affecting crown morphogenesis. However, increase in tooth size even after not significant increase in tooth size with mimic treatment implies that cell adhesion and actin remodelling may have impacted.

**Figure 3:**
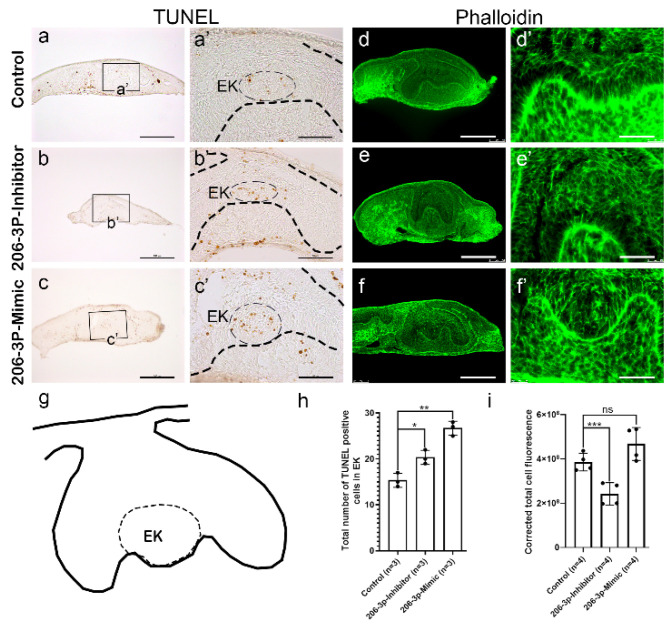
Cellular death and rearrangement after loss-and gain of function of *miR-206-3p*. Compared with the control (**a**–**a’**), TUNEL assay showed that epithelial and mesenchymal apoptosis were increased after inhibiting *miR-206-3p* (**b**–**b’**). Apoptosis especially increased in the EK after inhibiting *miR-206-3p* (**b’**,**h**). The mimic-treated specimen showed increased apoptosis in the epithelium, including EK, (**c**–**c’**) but not in the IEE. Cell rearrangement patterns were examined using phalloidin staining (**d**–**f**). Phalloidin staining intensity is decreased in the tooth organ after inhibiting *miR-206-3p* (**e**–**e’**,**i**) compared with that in the control (**d**–**d’**,**i**). However, the staining intensity was not alteredin the mimic-treated specimens (**f**–**f’**,**i**). Schematic of tooth organ with EK (**g**). EK; enamel knot. Boxes denote the magnified regions (**a**–**c**). Dotted lines indicate the epithelial boundary (**a’**–**c’**) and dotted ovals and or circles indicate the EK region (**a’**–**c’**,**g**). ns, *,** and *** indicate not significant, *p* < 0.05, *p* < 0.01 and *p* < 0.001, respectively. *Scale bars* 200 µm (**a**–**f**), 50 µm (**a’**–**f’**).

Meanwhile, epithelial and mesenchymal apoptosis increased after inhibition and or mimicking the *miR-206-3p* (*n* = 3) during in vitro organ cultivation (Figure 3b–b’,c–c’), compared to the control (Figure 3a–a’) (*n* = 3). In particular, apoptosis increased in the EK after treatment with inhibitor and mimic (Figure 3b’,c’,h), which further suggests that *miR-206-3p* have important role in EK morphogenesis that ultimately leads to crown morphogenesis. The final crown shape is determined by epithelial invagination and requires the changes in the cell motility and cell adhesion regulated by RAC1 and RHO-A [26], and can be evaluated by actin staining. The actin staining in the IEE (determined by phalloidin staining) was decreased after inhibiting *miR-206-3p* (Figure 3e–e’,i) compared with that in the control (Figure 3d–d’,i) (*n* = 4). Conversely, the actin staining intensity was not altered significantly in the IEE of mimic-treated specimens (Figure 3f–f’,i) (*n* = 4). At the same time, E-cadherin, an epithelial cell adhesion marker was reduced in the tooth epithelium after mimic treatment (Figure S2b) suggesting that *miR-206-3p* also regulate cell adhesion. 

### 2.3. miR-206-3p Modulates Tooth Specific Signaling 

**Figure 4:**
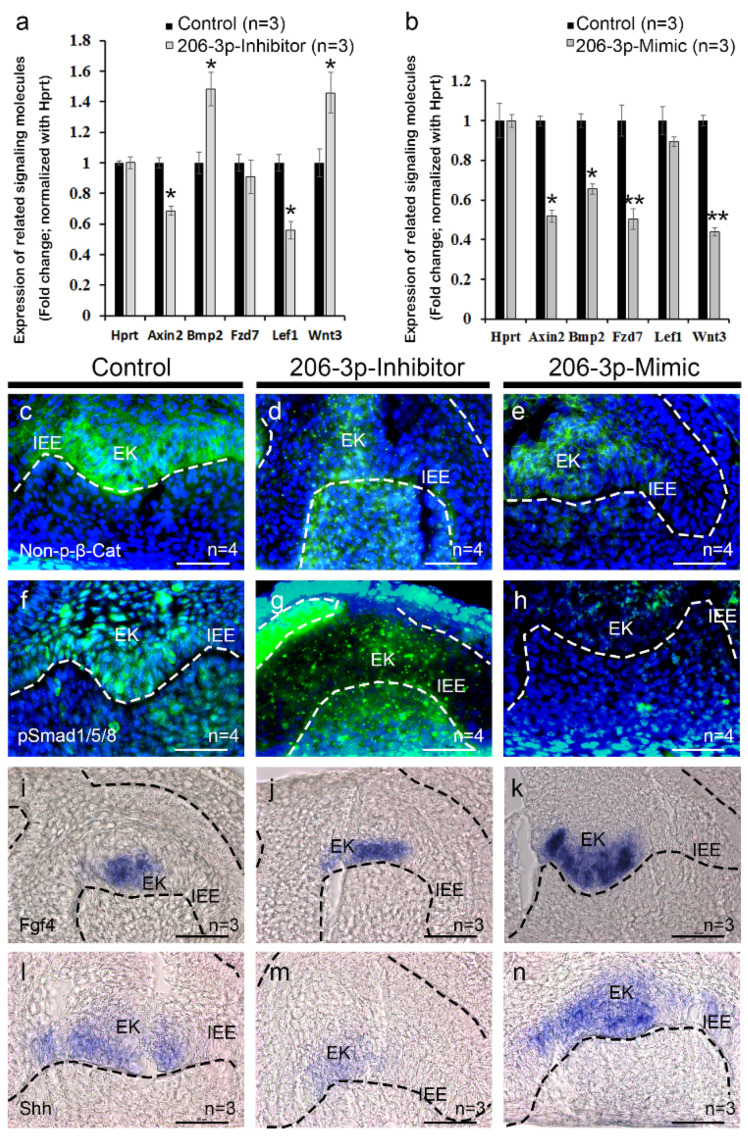
Altered signaling after loss or gain of function of *miR-206-3p*. Signaling factors related to tooth development were examined by qPCR (**a**–**b**). The expression of active β-catenin was reduced, and the localization patterns in the IEE and EK were destroyed after inhibiting *miR-206-3p* (**d**) compared with those in the control (**c**). However, a stronger mesenchymal expression of β-Catenin was observed (**d**). The specimen treated with the *miR-206-3p* mimic showed weaker expressions of β-catenin in the IEE and EK (**e**). The epithelial and mesenchymal expressions of pSMAD1/5/8 were reduced, and the localization patterns were impaired upon inhibition of *miR-206-3p* during the in vitro organ cultivation (**g**) compared with those in the control (**f**). The specimen treated with the mimic showed reduced expression of pSMAD1/5/8 in both, the epithelium and mesenchyme (**h**). FGF4 expression was detected within a restricted area in the EK of the control specimen (**i**). However, the expression was expanded in the IEE after inhibiting *miR-206-3p* (**j**). The specimen treated with the mimic showed an increased and expanded expression of *Fgf4* in the EK (**k**). Similarly, the *Shh* expression was decreased after the inhibition of *miR-206-3p* (**m**) compared with that in the control (**l**) but was increased in the specimens treated with the mimic (**n**). Dotted lines indicate the epithelial boundary (**c**–**n**). EK; enamel knot, IEE; inner enamel epithelium. Scale bars 50 µm (**c**–**n**). * and ** indicate not signigicant, *p* < 0.05 and *p* < 0.01, respectively. *Scale bars* 50 µm (**c**–**n**).

According to the target database TargetScan [27], *miR-206-3p* is broadly conserved among vertebrates and predicted to target *Wnt3* and *Fzd7* (Figure S3), important components of the canonical Wnt signaling and are also expressed during tooth development [4]. After successful transfection, expression levels of the genes related to tooth development were examined by qPCR (Figure 4a,b). The qPCR results showed that the *Wnt3* level was increased upon inhibition of *miR-206-3p* (Figure 4a). Meanwhile, the *Lef1* and *Axin2* levels were downregulated, and the *Bmp2* level was upregulated, whereas the *Fzd7* level was not significantly altered (Figure 4a). However, after treatment with *miR-206-3p* mimic, the *Wnt3, Fzd7, Bmp2,* and *Axin2* levels were significantly down-regulated (Figure 4b). To understand the altered expression patterns of signaling molecules in detail, we examined the localization of the proteins involved in the Wnt and Bmp signaling pathways, including active β-catenin and pSMAD1/5/8 in the cultivated teeth (Figure 4c–h). 

The β-catenin is reported to have role in tooth development from early placode to late bell stage [5] and tooth root formation [28] and nuclear β-catenin is used as an alternate readout for Wnt signaling activity [29]. In control (*n* = 4), the β-catenin was localized both in the nuclear and cytoplasmic regions of EK and adjacent cells in the mesenchyme (Figure 4c). In contrast, the protein level decreased, and its localization patterns were impaired in the IEE and EK after inhibiting *miR-206-3p* (Figure 4d) (*n* = 4), compared with those in the control (Figure 4c). However, a higher nuclear and cytoplasmic mesenchymal β-catenin level was observed (Figure 4d). Meanwhile, the mimic-treated specimen (*n* = 4) showed lower β-catenin levels in the IEE and EK when compared with the control (Figure 4e). The altered β-catenin localization in tooth suggests that Wnt signaling is affected, which can have further effect on other signaling regulation. 

During tooth development, Wnt and BMP signaling crosstalk in regulating the enamel knot morphogenesis and subsequent induction of BMP signaling in the mesenchyme [30]. Moreover, BMP signaling is transmitted through SMAD1 and 5 which are localized within the IEE and cranial neural crest derived dental mesenchyme at the cap and bell stage and regulate epithelial mesenchymal interaction during tooth morphogenesis [31]. To understand whether BMP signaling is altered after inhibition and mimicking the *miR-206-3p*, we used pSMAD1/5/8 immunohistochemistry. The epithelial and mesenchymal pSMAD1/5/8 levels were reduced, and the localization patterns were impaired after inhibiting *miR-206-3p* (*n* = 4) during the in vitro organ cultivation (Figure 4g), compared with those in the control (Figure 4f) (*n* = 4). The mimic-treated specimen showed reduced pSMAD1/5/8 levels in both the epithelium and mesenchyme (Figure 4h) (*n* = 4). 

To evaluate the role of *miR-206-3p* further in the morphogenesis of the enamel knot, we examined the expression of *Fgf4* and *Shh* by whole-mount or cryo-section in situ hybridization (Figure S4, Figure 4i–n). *Fgf4* was expressed within a restricted area in the EK of the control specimen (Figure 3i, Figure S4a) (*n* = 3). However, the expression expanded in the IEE after inhibiting *miR-206-3p* (Figure 4j, Figure S4b) (*n* = 3). The mimic-treated specimen (*n* = 3) increased and expanded the expression of *Fgf4* in the EK (Figure 4k, Figure S4c). Similarly, the *Shh* level was downregulated after the inhibition of *miR-206-3p* (Figure 4m, Figure S4e) relative to that in the control (Figure 4l, Figure S4d) but was upregulated in the mimic-treated specimens (Figure 4n, Figure S4f) (*n* = 3).

### 2.4. miR-206-3p Regulates Tooth Pulp Morphogenesis 

**Figure 5:**
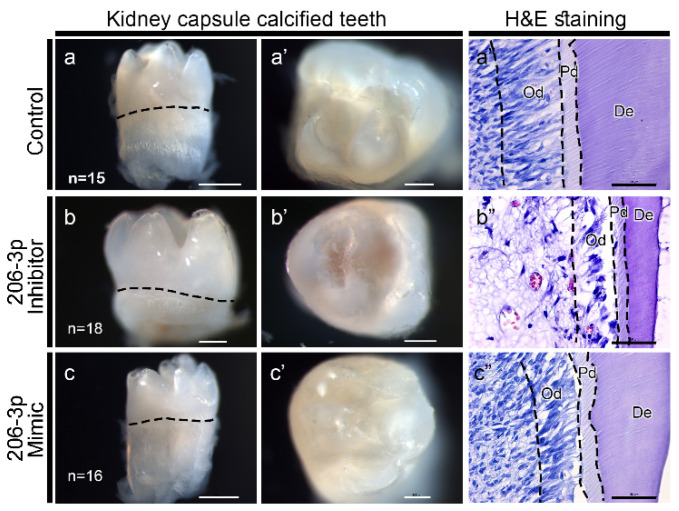
Renal-capsule–calcified teeth for 21 days (**a**–**c**). The buccal view of the teeth treated with the control (**a**), *miR-206-3p* inhibitor (**b**), or *miR-206-3p* mimic (**c**). The inhibitor-treated teeth are very small (**b**) relative to the control (**a**). Occlusal views showing the crown morphology (**a’**–**c’**). The histological sections of the calcified teeth show properly arranged odontoblast layer, predentin, and dentin in the control specimen (**a’’**). The inhibitor-treated specimen showed decreased cell mass in the pulp region with a poorly differentiated and developed odontoblast layer [(**b’’**) with thinner predentin and dentin layers (**b’’**)]. The mimic-treated specimen showed the proper amount of cellular mass in the dental pulp but irregularly arranged odontoblast, predentin, and dentin layers (**c’’**). Scale bars 500 µm (**a**,**e**), 200 µm (**a’**,**b**–**b’**,**c’**), 50 µm (**a’’**–**c’’**).

To identify the role of *miR-206-3p* in tooth development, we examined renal-capsule–calcified teeth (Figure 5a–c). The inhibitor-treated teeth (*n* = 18) were very small after 21 days of calcification in the renal capsule (Figure 5b–b’, Figure S5b) relative to the control (*n* = 15) or mimic-treated teeth (*n* = 16) (Figure 5a–a’,c–c’, Figure S5a,c). The histological examination of the calcified teeth showed a properly arranged odontoblast layer, predentin, and dentin in the control specimen (Figure 5a”, Figure S5a). Meanwhile, the inhibitor-treated specimen decreased in cell mass in the pulp region, and had a poorly differentiated and developed odontoblast layer with thinner predentin and dentin layers, relative to those in the control (Figure 5b”, Figure S5b). However, the mimic-treated specimen showed a proper amount of cell mass in the dental pulp, but the arrangement of the odontoblast layer was irregular and also showed irregular predentin and dentin layers (Figure 5c”, Figure S5c). These observations suggest that signaling modulation, mediated by *miR-206-3p,* is crucial for tooth crown and pulp morphogenesis.

## 3. Discussion

Signaling regulation mediated by epithelial-mesenchymal interactions during tooth development is crucial to form optimal tooth size with proper crown and pulp development [30]. The Wnt is one of the important signaling in tooth development and has been extensively studied [5]. Here, we report that *miR-206-3p* in tooth development primarily regulates Wnt signaling that mediate other signaling such as BMP, SHH and FGF for proper morphogenesis of tooth crown and pulp. 

The stage-specific expression pattern of *miR-206-3p* in the dental epithelium and mesenchyme at the bud stage, and restricted expression in the epithelium at the later stages of tooth development coincided with the expression patterns of other signaling factors, including *β-catenin* [5], *Lef1* [7], *Pitx2* [32] and *Wnt3* [4]. This coincided expression pattern suggested that *miR-206-3p* would have major roles in modulating signaling factors related with tooth development. Accordingly, we examined the precise role of *miR-206-3p* in tooth development by employing in vitro organ cultivation of embryonic tooth using an inhibitor or mimic of *miR-206-3p*. 

In this research we found that *miR-206-3p* controls tooth size by regulating the cell dynamics including cell adhesion, proliferation, apoptosis and cytoskeletal remodelling. Our results showed that cell proliferation still remained in the EK after inhibiting and mimicking the *miR-206-3p,* suggesting that there is decrease in cell adhesion in the epithelium, which later affects the signaling between IEE and dental papilla. At the same time, apoptosis increased in the EK after inhibiting and mimicking the *miR-206-3p,* which seems unusual because proliferation is also persistent in the EK. These data suggest that tooth EK tends to acquire the proper tooth morphology by limiting the numbers of proliferative cells in the EK. In zebrafish study, both the overexpression and knockdown of *miR206* impaired the actin filament formation during gastrulation [33]. Moreover, developing tooth maintains its morphology through actin fibers, regulated by Rho-associated *protein* kinase (ROCK) and important for proper ameloblast and odontoblast formation [34]. The inhibition of *miR-206-3p* reduced the actin reorganization suggesting that a more pronounced changes results in the tooth crown and pulp formation. As reported in a previous study, the mesenchymal cell number is an important factor for tooth size determination [22], which suggest that, in our study, a reduced number of proliferative cells in the mesenchyme after treatment with inhibitor, also contributed to significant reduction of tooth size (Figure 2e). Meanwhile, reduced localization of E-cadherin in the tooth organ (Figure S2) might be responsible for increased size of tooth germ in the mimic treated specimen, due to reduced adhesion as the reduction in E-cadherin is associated with the loss of contact inhibition and increased cell motility and proliferation [35]. Our observations provide strong evidence for the involvement of *miR-206-3p* in EK morphogenesis and regulation of gene expression, as previously reported [36]. 

Epithelial-mesenchymal interactions are important throughout tooth development [37]. Moreover, previous reports have implicated that EK serves as the primary signaling center during tooth development [38], and altered EK affects the functional structure of the tooth, particularly the cusp pattern [39]. *Shh* expressed in the epithelium regulates *Bmp* s in the mesenchyme, which regulates *Dspp* during terminal differentiation of odontoblasts [40,41]. Loss of *Lef1* causes arrested tooth development at bud stage, loss of expression of LEF1/β- catenin target gene *Fgf4* and failure of survival of dental epithelial cells [7,42]. *Bmp2* is required for odontoblast differentiation and pulp vasculogenesis [43]. Beta catenin enhances odontoblastic differentiation in pulp cells through regulation of *Runx2* [44]. Inhibition of *miR-206-3p* results in reduced expression of *Lef1* and *Axin2,* and increased the expression of *Bmp2* and *Wnt3* suggesting the crosstalk of Bmp and Wnt signalings. miR-*206*-3p regulates Wnt signaling, as previously predicted by the TargetScan database [27], further suggesting that loss of *miR-206-3p* regulate Bmp signaling indirectly via Wnt signaling. Meanwhile, expression pattern of β-catenin, the mediator of the Wnt signalling, was upregulated after the inhibition of *miR-206-3p* and would alter the fate of tooth [45]. 

Conversely, we found that, *Axin2*, *Bmp2*, *Fzd7* and *Wnt3* were downregulated in mimic-treated specimens and showed disrupted localization patterns of β-catenin and pSMAD1/5/8 further suggests that *miR-206-3p* also regulates the Bmp signaling through the regulation of the Wnt signaling. Down-regulation of *Axin2* both in the inhibitor and mimic treated specimen suggested that *Axin2* functions autonomously in treated tissues not working as a negative feedback regulator of Wnt signaling. Further, the levels of *Fgf4* and *Shh*, two important signaling factors for tooth development [46,47], were altered in the EK and IEE, presumably contributing to the altered morphogenesis of EK and overall tooth structure. These results suggested that *miR-206-3p* regulated tooth development through the Wnt, Bmp, and Shh signaling pathways. However, further analyses using Wnt antagonists and activators are needed for confirmation.

Calcified teeth by renal capsule transplantation revealed that *miR-206-3p* was indispensable during tooth development. It has previously been reported that the Shh signaling in the epithelium signals the mesenchyme to induce the Bmp signaling during the differentiation stages [43,48]. This observation suggests that differentiation during tooth development was altered after treatment with the *miR-206-3p* inhibitor or mimic in the current study. Moreover, extra-folding of IEE, altered ameloblast differentiation, and defective enamel formation in the *Dicer-1* conditional knockout mouse suggests that fine-tuning of gene expression is necessary for proper hard-tissue formation [49]. In addition, other recent studies have suggested that *miRNA 200c/141* knockout mice are defective in enamel formation through changes in the Bmp signaling [50]. In this study, altered epithelial and mesenchymal Wnt signaling upon modulation of the *miR-206-3p* levels resulted in changes in the downstream signaling during tooth development, leading to altered tooth formation. Thus, *miR-206-3p* appears to be a pivotal factor regulating tooth formation by targeting the Wnt signaling.

Based on our results, we conclude that miRNA–mediated modulation of the Wnt signaling is necessary for proper tooth morphogenesis through tight regulation of tooth developmental processes. Disruption of miRNA balance would lead to significant alterations in tooth morphology including tooth size and hard tissue matrices formation. A better understanding of the fine-tuning of tooth developmental signaling pathways, through single miRNA modulation during tooth development is needed to understand the etiopathology of dental defects in humans.

## 4. Materials and Methods

All experimental protocols were approved by the Kyungpook National University, School of Dentistry, Animal Care and Use Committee (KNU-2012-42, 3 April 2012) and performed according to the ARRIVE guidelines for the care and use of laboratory. 

### 4.1. Animals

Adult ICR (Institute for Cancer Research) mice were housed in a temperature-controlled room (22 °C) under artificial illumination (lights on from 05:00 to 17:00) and 55% relative humidity with access to food and water ad libitum. The mouse embryos were obtained from time-mated pregnant mice. The day on which a vaginal plug was confirmed were designated as embryonic day 0 (E0). The embryos at E13, E14, and E15 were used. 

All experiments described in this study were performed for 3 or more times, independently.

### 4.2. In Situ Hybridization 

For miRNA in situ hybridizations, a 5’- and 3’- DIG-labeled miRCURY LNA^TM^ miRNA custom detection probe at 80 nM final concentration was hybridized at 54 °C using the miRNA ISH buffer set (Exiqon, Skelstedet, Denmark, cat no. 90000) and processed according to the manufacturer’s instructions. The miRCURY LNA^TM^ miRNA custom detection probe for *miR-206-3p* (/5’DigN/CCACACACTTCCTTACATTCCA/3’Dig_N/) was obtained from Qiagen (Qiagen, Hilden, Germany, cat. no. 339115 YCD0072334-BCG). Whole-mount or section in situ hybridizations were performed, as previously described [51], using standard protocols in RNAse free conditions. Briefly, for section in situ hybridization, 7 µm thick tissue sections were deparaffinised and rehydrated in PBS and treated with proteinase K for 15 min. The sections were postfixed in 4% PFA and processed for acetylation using mixture of acetic acid anhydride and triethanolamine. For whole mount in situ hybridization, the tissues were rehydrated using methanol and treated with proteinase K. The tissue were then postfixed with mixture of glutraldehyde and paraformaldehyde and prehybridized in hybridization solutions. For both hybridizations, digoxigenin (DIG)-labeled antisense RNA probes were pre-warmed to 80 °C and hybridized overnight at 62 °C. After whole-mount in situ hybridization, 20-μm thick frontal frozen sections were prepared to examine the detailed expression patterns.

### 4.3. In Vitro Organ Cultivation and Renal Capsule Transplantations

The embryonic mice molar tooth buds were micro-dissected out from the lower jaws of E13 mice in PBS under a stereo-microscope. The tooth buds were cultured in DMEM (HyClone, Logan, UT, USA; cat. no.-SH30243.01) with 10% fetal bovine serum (Hyclone, Logan, UT, USA) and antibiotics using a modified Trowell’s culture method for a two days, as previously described [52]. During the cultivation, the tooth germs were transfected with 400 nM of the inhibitor or mimic of the *miR-206-3p* as previously described [53]. The inhibitor and mimic were purchased from Qiagen (Qiagen, Hilden, Germany, miCURY LNA^TM^ miRNA Power Inhibitor, cat no. 339131 YI04100975-DDB; miCURY LNA^TM^ miRNA Mimic, cat no. 339173 YM00472240-ADB). The cultivated tooth germs were transplanted into the renal subcapsular layer of the adult male mice as previously described [22]. After 3 weeks, the host mice were sacrificed, and kidneys were dissected out to obtain the calcified teeth.

### 4.4. Histology and Immunohistochemistry

Immunostaining and routine histological analyses by H and E staining were carried out, as previously described [51]. Calcified teeth were sectioned following decalcification using 0.5 M EDTA for three weeks. For immunostaining, sections were first rehydrated and then processed for antigen retrieval. Blocking was performed by incubating the sections in the 1X western blocking solution (Roche, Mannheim, Germany; Ref. 11921673001) for one hour at room temperature. The primary antibodies used in this study were directed against Ki67 (Thermo Scientific, Waltham, MA, USA, cat. no. RM-9106-s), non-phospho β-catenin (Cell Signaling Technology, Danvers, MA, USA, cat. no. 8814S), and pSMAD (Cell Signaling Technology, Danvers, MA, USA; cat. no. 9511S). The secondary antibodies used in this study were biotinylated goat anti-rabbit IgG (Invitrogen, Waltham, MA, USA) and goat anti-rabbit IgG Flamma 488 (BioActs, Incheon, Korea, cat. no. RSA1241). 

### 4.5. TUNEL Assay

TUNEL assay was performed as previously described [54] using an in situ cell apoptosis detection kit (Trevigen, Gaithersburg, MD, USA, cat. no. 4810-30-K) according to the manufacturer’s instructions.

### 4.6. Phalloidin Staining 

Phalloidin staining was performed as previously described [54]. Frozen sections were incubated with phalloidin-fluorescein isothiocyanate (Sigma-Aldrich, St. Louis, MO, USA, Cat no. p5282; Sigma) at room temperature for 1 h, and then visualized under a fluorescence microscope DM2500 (Leica, Wetzlar, Germany). 

### 4.7. Quantitative PCR (qPCR)

RNA was extracted from cultivated tooth germs using the RNeasy micro Kit (Qiagen, Hilden, Germany, cat. no. 74004), and qPCR was carried out, as previously described [51]. For miRNA qPCR, RNA was extracted from the cultivated teeth using the miRNeasy Micro Kit (Qiagen, Hilden, Germany, cat. no. 217084), and cDNA was prepared using the miScript II RT Kit (Qiagen, Hilden, Germany, cat. no. 218161). The primers for miRNA qPCR were designed using miRprimer [55]. The mRNA and the miRNA qPCR results for each sample were normalized to those of *Hprt* and *snoRNA135*, respectively. The data are expressed as means ± standard deviations (SDs). The mean expression levels were compared between the experimental and control groups using Student’s *t*-test. *p*-values < 0.05 were considered significant. The primers used in this study are presented in Table 1. This experiment is repeated for 3 independent biological samples. 

## Figures and Tables

**Table 1:** Primer sequences for qPCR.

Gene	Accession	Primer Sequence		References	Product Size (bp)	Remark
*Axin2*	BC057338.1	Forward	TGAAGAAGAGGAGTGGACGT	[56]	115	Wnt signaling
		Reverse	AGCTGTTTCCGTGGATCTCA			
*Bmp2*	NM_007553.3	Forward	AAGTGGCCCATTTAGAGGAG	[56]	104	Bmp signaling
		Reverse	CAATGGCCTTATCTGTGACC			
*Fzd7*	NM_008057.3	Forward	AAGGGGGAAACTGCGGTATG		203	Wnt Signaling
		Reverse	TCAAAACCATCTCTCGCCCC			
*Lef1*	NM_010703.4	Forward	ACAGCGACGAGCACTTTTCT	[56]	82	EK signaling
		Reverse	TGTCTGGACATGCCTTGCTT			
*Wnt3*	NM_009521.2	Forward	TCGAGGCTTACCTTTGCACAT		114	Wnt signaling
		Reverse	TGGTCTTGTCCTTCCCACCA			
*Hprt*	NM_013556.1	Forward	CCTAAGATGATCGCAAGTTG	[56]	86	Internal standard
		Reverse	CCACAGGGACTAGAACACCTGCTAA			
**Primer Sequences for miRNA qPCR**						
**Gene**	**Accession**	**Primer Sequence**				
*miR-206-3p*	MI0000249 (miRBase)	Forward	GCAGTGGAATGTAAGGAAGT			
		Reverse	CCAGTTTTTTTTTTTTTTTCCACACA			
*snoRNA135*	AF357323 (NCBI)	Forward	TGGAATTACCGGCAGATTGGTAGTGGTGAGCCTATGGT			
		Reverse	TCCAGTTTTTTTTTTTTTTTCTTCAGA			

## Supplementary Materials

The following are available online at https://www.mdpi.com/1422-0067/21/15/5251/s1. Figure S1. Evaluation of the efficiency of inhibitor/mimic transfection during in vitro organ cultivation. Figure S2. Localization of E-Cadherin in E13+2 day teeth. Figure S3. The putative binding sites of mmu-miR-206-3P are broadly conserved among the Wnt3 and Fzd7 3’ UTRs of vertebrates. Figure S4. Whole-mount in situ hybridization. Figure S5. Lower magnification of H&E-stained histological sections of the renal-capsule–calcified teeth.
