# Supplementary material for: Signaling Modulations of miR-206-3p in Tooth Morphogenesis
Source: Int J Mol Sci. 2020 Jul 24;21(15):5251. doi: 10.3390/ijms21155251 (PMC7432545; doi:10.3390/ijms21155251)
Supplement: Supplementary file 1 [file ijms-21-05251-s001.pdf]

Signaling modulations of miRNA 206-3p in tooth morphogenesis

Sanjiv Neupane[1, 6*], Yam Prasad Aryal[1], Tae-Young Kim[1], Chang-Yeol Yeon[1], Chang-Hyeon An[2], Ji-Youn Kim[3], Hitoshi Yamamoto[4], Youngkyun Lee[1], Wern-Joo Sohn[5], Jae-Young Kim[1*]

[1]Department of Biochemistry, School of Dentistry, Kyungpook National University, Daegu, Korea; sanjiv.knu@gmail.com (S.N.); yamaryal@yahoo.com (Y.P.A.); tae09290@daum.net (T.Y.K.); yhs2669@naver.com (C.Y.Y); jykim91@knu.ac.kr (J.Y.K)

[2]Department of Oral and Maxillofacial Radiology, School of Dentistry, Kyungpook National University, Daegu, Korea; chan@knu.ac.kr

[3]Department of Dental Hygiene, College of Health Science, Gachon University, Incheon, Korea; hoho6434@gachon.ac.kr

[4]Department of Histology and Developmental Biology, Tokyo Dental College, Tokyo, Japan; hyamamoto@tdc.ac.jp

[5]Pre-Major of Cosmetics and Pharmaceutics, Daegu Haany University, Gyeongsan, Korea; undine75@gmail.com

[6]Department of Biochemistry and Cell Biology, Stony Brook University, 450 Life Science Building, 370 Center for Molecular Medicine, Stony Brook, NY 11794-5215; sanjiv.neupane@stonybrook.edu

Correspondence:

Jae-Young Kim, Ph.D.,

Department of Biochemistry, School of Dentistry, IHBR, Kyungpook National University, 2177 Dalgubeol-daero (41940), Jung-gu, Daegu, Republic of Korea

Tel: 82-53-420-4998, Fax: 82-53-421-4276, E-mail: jykim91@knu.ac.kr

Sanjiv Neupane, Ph.D.

Department of Biochemistry, School of Dentistry, IHBR, Kyungpook National University, 2177 Dalgubeol-daero (41940), Jung-gu, Daegu, Republic of Korea

Email: sanjiv.knu@gmail.com, sanjiv.neupane@stonybrook.edu

Current address: Department of Biochemistry and Cell Biology, Stony Brook University, 450 Life Science Building, 370 Centers for Molecular Medicine, Stony Brook, NY 11794-5215

Supplementary materials

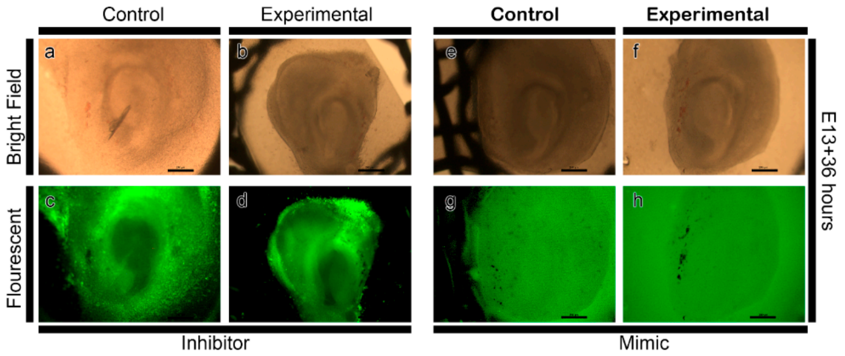

Supplementary Figure S1. Evaluation of the efficiency of inhibitor/mimic transfection during *in vitro* organ cultivation

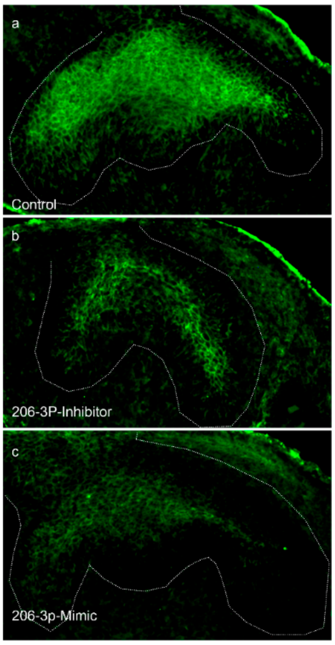

Supplementary Figure S2. Localization of E-Cadherin in E13+2 day teeth

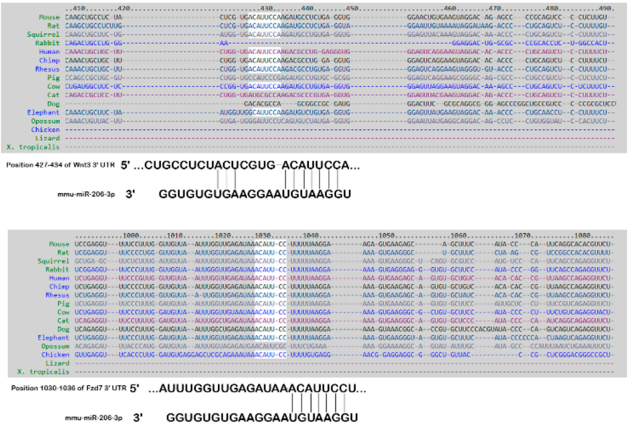

Supplementary Figure S3. The putative binding sites of mmu-miR-206-3P are broadly conserved among the Wnt3 and Fzd7 3′ UTRs of vertebrates.

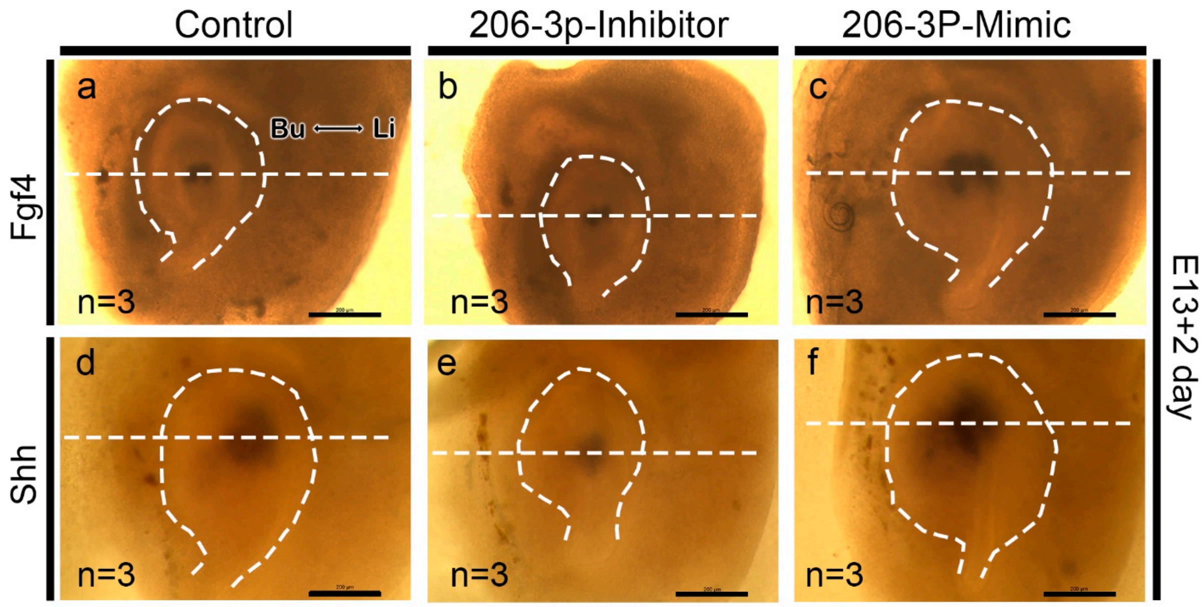

Supplementary Figure S4. Whole-mount *in situ* hybridization. Whole-mount in situ hybridization of *Fgf4* (a-c) and *Shh* (d-f) in E13 + 2 day–cultivated teeth (d-f). Dotted lines indicate the levels of the sections presented in Figure 3. The boundaries of tooth germs are indicated by the dotted lines (white). Bu; buccal, Li; lingual. *Scale bars* 200 μM.

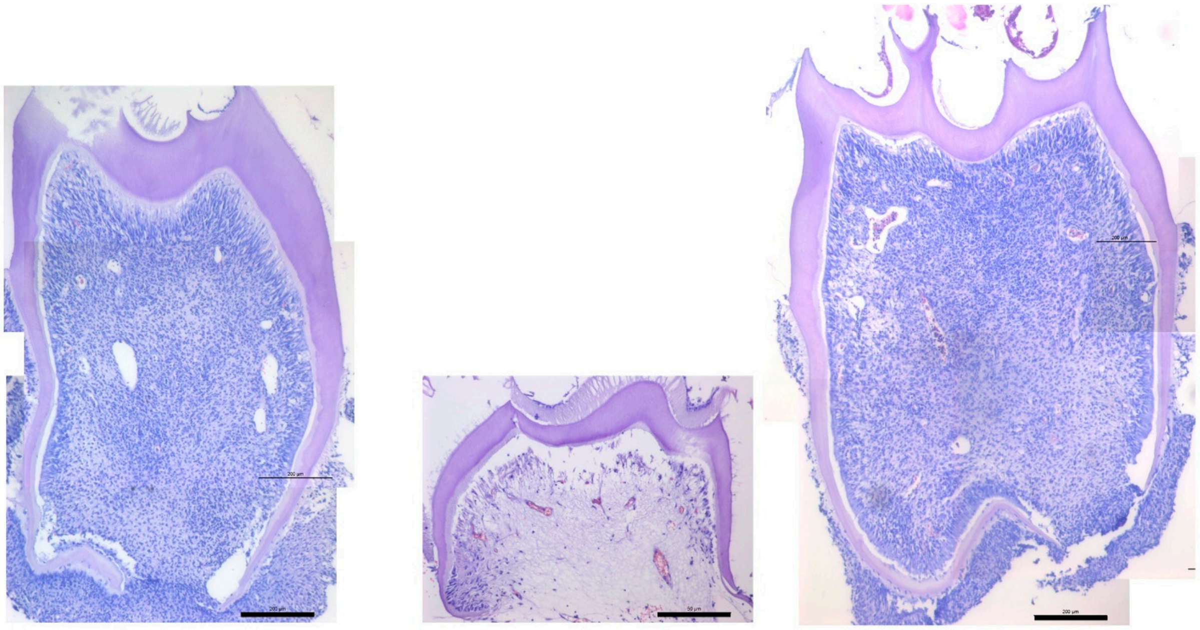

Control 206-3P-Inhibitor 206-3P-Mimic

Supplementary Figure S5. Lower magnification of H&E-stained histological sections of the renal-capsule–calcified teeth. *Scale bars* 200 μM
